# Developing an Unbiased Multiplex PCR System to Enrich the *TRB* Repertoire Toward Accurate Detection in Leukemia

**DOI:** 10.3389/fimmu.2020.01631

**Published:** 2020-08-06

**Authors:** Jinghua Wu, Xie Wang, Liya Lin, Xuemei Li, Sixi Liu, Wei Zhang, Lihua Luo, Ziyun Wan, Mingyan Fang, Yi Zhao, Xiaodong Wang, Huirong Mai, Xiuli Yuan, Feiqiu Wen, Changgang Li, Xiao Liu

**Affiliations:** ^1^BGI Education Center, University of Chinese Academy of Sciences, Shenzhen, China; ^2^BGI-Shenzhen, Shenzhen, China; ^3^Hematology and Oncology Department, Shenzhen Children's Hospital, Shenzhen, China; ^4^Department of Computer Science, City University of Hong Kong, Hong Kong, China; ^5^Neoimmune, Shenzhen, China

**Keywords:** immune repertoire, *TRB*, multiplex PCR system optimization, minimal residual disease, leukemia

## Abstract

Accurate T cell receptor repertoire profiling has provided novel biological and clinical insights in widespread immunological settings; however, there is a lack of reference materials in the community that can be used to calibrate and optimize the various experimental systems in different laboratories. In this study, we designed and synthesized 611 T cell receptor (TCR) beta chain (*TRB*) templates and used them as reference materials to optimize the multiplex PCR experimental system to enrich the *TRB* repertoire. We assessed the stability of the optimized system by repeating the experiments in different batches and by remixing the TRB templates in different ratios. These TRB reference materials could be used as independent positive controls to assess the accuracy of the experimental system, and they can also be used as spike-in materials to calibrate the residual biases of the experimental system. We then used the optimized system to detect the minimal residual disease of T cell acute lymphoblastic leukemia and showed a higher sensitivity compared with flow cytometry. We also interrogated how chemotherapy affected the TCR repertoire of patients with B-cell acute lymphoblastic leukemia. Our result shows that high-avidity T cells, such as those targeting known pathogens, are largely selected during chemotherapy, despite the global immunosuppression. These T cells were stimulated and emerged at the time of induction treatment and further expanded during consolidation treatment, possibly to fight against infections. These data demonstrate that accurate immune repertoire information can improve our understanding of the adaptive immunity in leukemia and lead to better treatment management of the patients.

## Introduction

The human T cell repertoire is generated by somatic rearrangement of the variable (V), diversity (D), and joining (J) segments on the T cell receptor (TCR) loci during T cell development, and a diverse TCR repertoire is pivotal to human health. Before the wide application of high throughput sequencing (HTS), spectratyping assays was the commonly used method for assessing the TCR repertoire. However, TCR spectratyping is limited by its low resolution because it cannot provide precise information on the individual clonotype (1–3). The application of HTS in TCR repertoire analysis (TCR-seq) began 10 years ago (4–6) and has been widely used in basic and translational research (2, 7–9) due to its high resolution and throughput. The technology has been used in fields such as investigating the functional heterogeneity of the human T cell (10), comparing TCR repertoire of different tissues (11, 12), analyzing the effect of genetic background on TCR repertoire (13), identifying autoimmune-disease-related T cells (14–16), quantifying minimal residual disease in blood cancer (17), and predicting the responses of vaccination (18) or immunotherapy (19–21).

Single cell sequencing approach (e.g., 10X genomics) can provide information of α chain and β chain pairing, as well as transcription information of other genes in TCR repertoire study. However, it is still relatively expensive at present, and fresh specimens are usually required for living single cell isolation, which limit its broad application in disease and health research (22). In large cohort study, sequencing TCR repertoire from bulk cells is more commonly used because of its low cost, high throughput, and easy access to samples. For bulk TCR-seq, both genomic DNA and RNA can be used as the input material. The consistent copy number (only one productively rearranged alpha locus and beta locus per αβ T cell) and the accessibility make genomic DNA (gDNA) more convenient in future clinical applications.

Multiplex polymerase chain reaction (PCR) and rapid amplification of 5′ complementary DNA ends (5′RACE) are the two most commonly used library preparation methods. Although 5′RACE can control bias by refraining from the use of multiple primer sets, it works only for RNA (2, 22). Multiplex PCR was used as the most efficient and cost-effective method to enrich the completely rearranged TCR genes of both DNA and RNA samples. However, multiplex PCR can introduce biases due to differences in amplification efficiencies and cross-reactivities of multiple primers in the same reaction system, which can compromise the precision of the TCR repertoire data. For RNA sample, unique molecular identifiers (UMIs) can be incorporated by reverse transcription to adjust the bias (23, 24); however, it is challenging to bring in UMIs for gDNA sample. Therefore, it is critical to optimize the multiplex PCR experimental system to minimize the biases. In a previous effort, Carlson et al. (25) synthesized 56 TCR gamma chain *(TRG)* template sets to optimize the TRG repertoire amplification system; however, for the amplification system of other TCR chains, especially the most diverse β chain, few have been carefully refined in controlled systems or by reference standard materials.

Increased T cell infiltration has been widely reported to be associated with improved survival in patients with solid tumors (26–29). For blood cancer, the association between increased T cell infiltration with improved clinical outcome has been observed in diffuse large B-cell lymphoma (DLBCL) (30, 31). Beside the T cell infiltration level, Keane et al. (32) found that the clonality of intratumoral TCR repertoire is also associated with the clinical outcome of DLBCL (32). The T cells are dysfunctional in chronic lymphocytic leukemia (CLL), which is related with increased risk of infectious and autoimmune disease (33–35). Moreover, the TCR repertoire is skewed, which is reflected by restricted TRBV usage or increased clonality in CLL patients (36, 37). During ibrutinib therapy of CLL patients, large numbers of new T cell clonotypes emerged, and the TCR repertoire diversity increased, which may be related with decreased infection and improved clinical outcome (38). Chemotherapy is the mostly used treatment for acute lymphoblastic leukemia (ALL). Given the importance of T cells in fighting infections, the reconstruction of T cell immune repertoire during chemotherapy is very critical for the recovery from disease. In this study, we have designed a reference standard to develop an optimal reaction system for TCR beta chain (*TRB*) amplification, and then, we used this system to analyze the changes in *TRB* repertoire in acute ALL patients during chemotherapy.

## Methods

### Synthesis of *TRB* Templates

We designed 611 *TRB* templates with known sequences, and then, those templates were synthesized and cloned into the pUC57-simple plasmid vector to produce a recombinant plasmid DNA. Then, the recombinant plasmid DNAs were transformed into *Escherichia coli* for sequencing and long-term storage. The concentration of each recombinant plasmid DNA containing synthetic *TRB* templates was determined using the Qubit 3.0 fluorometer (Life Technologies, Paisley, UK). Then, we pooled all the recombinant plasmid DNA at expected equimolar levels, and the pooling was repeated twice.

### Quantifying the Composition of the Preamplified *TRB* Templates

To quantify the frequencies of each *TRB* template in the pool, the recombinant plasmid DNA pool was digested by restriction enzymes, and the synthetic *TRB* templates were purified and separated from the plasmid vector by gel extraction. Then, *TRB* templates were ligated with sequencing adapter and sequenced on BGISEQ-500 platform.

### Enriching the Rearranged *TRB* Segments by Multiplex PCR

Two steps of PCR were used to enrich the complete rearranged *TRB* fragments. For the pooled recombinant plasmid DNAs, 100,000 templates were used, and for the natural human samples, 300–1,200 ng DNA was used. The first step PCR is a multiplex PCR, and it goes 30 cycles, which includes 28 forward primers located at the *TRBV* FR3 regions and 13 reverse primers located at the *TRBJ* regions (Table S1). The second step PCR is a simplex PCR using a universal primer and goes seven cycles, which brings in the whole adaptor sequence for BGISEQ-500 platform to generate sequencing libraries.

### Sequencing and Data Analyzing

The sequencing libraries were used to make the DNA nanoballs and then sequenced on BGISEQ-500 platform with a single-end 200-bp reads. The raw sequencing data of the pre- and post-amplified synthetic *TRB* templates were aligned to the 411 *TRB* references using Bowtie 2 with local mode. For the natural human samples, the raw sequencing reads were processed using *IMonitor* (39), and the CDR3s in a sample with the frequency of <3 in 1 million sequences were filtered to remove the CDR3s containing sequencing errors.

### Optimizing Mixing Ratios of the Primers

Firstly, the V and J primers were mixed equimolarly, and the amplification bias of each V or J segment was calculated as the following formula:

Bias = (the V or J represent of postamplified templates)/(the V or J represent of preamplified templates).

For each V or J primer, if the amplification bias is >2 or <0.5, the proportion of the primer in the primer mixture will be adjusted until the amplification biases of most primers are in the range of 0.5–2.

### Minimal Residual Disease Detection and the Repertoire Characteristics Analysis in ALL Samples

We enrolled 10 childhood patients with B-cell acute lymphoblastic leukemia (B-ALL) and two childhood patients with T cell acute lymphoblastic leukemia (T-ALL) in Shenzhen Children's Hospital. The study was carried out in accordance with the recommendations of Declaration of Helsinki. It was approved by BGI Institute of Review Board (BGI-IRB) and written informed consent was obtained from the parent(s) or guardian(s) of each patient. The clinical information of the patients is shown in Table S2. The bone marrow (BM) specimens were obtained at diagnosis and on day 33 and 64 during chemotherapy, and 30 BM samples were collected in total. The BM samples were stored at −80°C, and the genomic DNA was extracted using a DNA Blood Mini Kit (QIAGEN, Cat. no. 51106).

The minimal residual disease (MRD) detection in T-ALL patients was conducted following the method introduced in our previous paper (40). All the TRB clonotypes in this paper refer to the CDR3 sequences. Due to the effect of reads number on the repertoire diversity, only 0.5 million effective reads were used when calculating the Shannon index, Pielou's index, and top 100 clonotype frequency. The Shannon index was calculated using the formula described in a previous paper (39); Pielou's index was calculated using the following forum: Pielou's index = Shannon index/ln *S* (*S* is the unique clonotype number of a repertoire). The proportion of known pathogen-specific *TRB* clonotypes (PKPSC) was calculated using the method described before (41).

### Statistics

Statistical analysis and data visualization were performed using R tools. The statistical significance of group comparison was tested using Wilcoxon rank sum test, and paired test was used where possible.

## Results

### Design of Reference Standard Materials to Minimize the Amplification Bias

The human *TRB* locus encodes 48 functional *TRBV* segments and 13 functional *TRB*J segments in IMGT. However, due to the germline sequence of *TRBV6-2* and *TRBV6-3* being completely identical, there are 47 different functional *TRBV* segments. Therefore, we designed 611 (47 × 13) synthetic *TRB* templates in total to simulate all the possible random rearrangement pairs of *TRBV* and *TRB*J segments. Those templates contain the germline sequence of the whole *TRBV* segments, a simulated complementarity determining region 3 (CDR3) with a length of 43 bp, the germline sequence of framework region 4 (part of J segments), and part of C segments.

**Figure 1:**
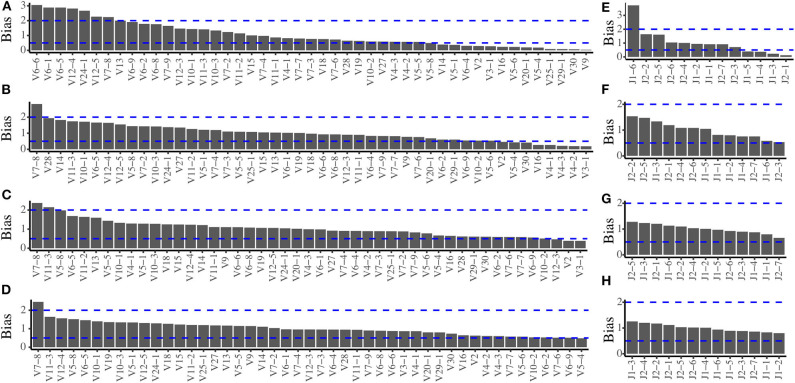
Amplification bias of the multiplex PCR reaction system during primer mix optimization for pool 1. **(A)** The *TRBV* amplification bias before optimization. **(B,C)** The *TRBV* amplification bias during optimization. **(D)** The *TRBV* amplification bias after optimization. **(E)** The *TRBJ* amplification bias before optimization. **(F,G)** The *TRBJ* amplification bias during optimization. **(H)** The *TRBJ* amplification bias after optimization.

In order to evaluate the amplification bias of the multiplex PCR system, we pooled all the *TRB* templates at expected equimolar levels and two repeated pools (pools 1 and 2) were generated. The actual frequency of each of the 611 *TRB* templates of the two pools before amplification is quantified by sequencing (Figure S1), and the actual usage frequency of each *TRBV* segments and *TRBJ* segments before amplification are shown in Figure S2. After amplification by multiplex PCR, we calculated the *TRBV* and *TRBJ* segments usage frequencies using the same methods and compared them with the frequencies before amplification to assess the amplification bias. In pool 1, when the primers were mixed at equimolar, there are severe amplification biases for both the *TRBV* (Figure 1A) and *TRBJ* segment (Figure 1E) before any experimental optimizations. We then adjusted the ratio of each primer in the primer mixture, and the results showed a less difference between the pre- and postamplified templates (Figures 1B,C,F,G). After several rounds of adjustments, the amplification biases for most of the primers were in the range of 0.5–2 for both *TRBV* segments (Figure 1D) and *TRBJ* segments (Figure 1H). The result of pool 2 showed similar results with pool 1 (Figure S3).

### The Stability of the Optimized Reaction System

**Figure 2:**
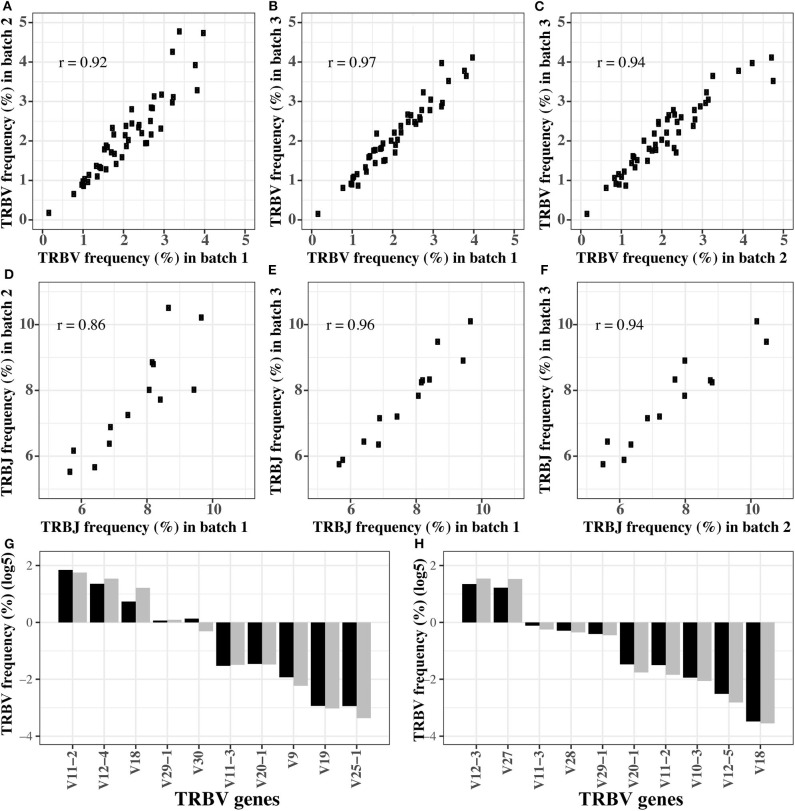
The robustness of optimized multiplex PCR reaction system. **(A–C)** The *TRBV* usage frequency distributions in three batches of experiments. **(D–F)** The *TRBJ* usage frequency distributions in three batches of experiments. **(G,H)** The *TRBV* usage frequency before and after amplification for the first **(G)** and second **(H)** gradient mixture of the *TRB* templates. In **(G,H)**, the black bar is the TRBV usage before amplification, and gray bar is the TRBV usage after amplification.

In order to verify the stability of the optimized reaction system, we replicated the amplification of the pooled *TRB* templates (pool 1) in three different batches using the final primer mix. The Pearson coefficient of *TRBV* segment usage was 0.92, 0.94, and 0.97, respectively, between any two of the three batches (Figures 2A–C). For *TRBJ* segments usage, the Pearson coefficient was 0.86, 0.96, and 0.94, respectively, between any two of the three batches (Figures 2D–F). These results showed that the reaction system is stable and is minimally affected by experimental batches or operations.

### Evaluating the Robustness of the Optimized Reaction System by Gradient Mixture

In order to test if the reaction system is robust when the templates are pooled in different concentrations, we remixed the 611 *TRB* templates at different concentrations to produce two new templates pools, and the expected concentration of each *TRB* template is shown in Tables S3, S4. Then, the preamplified pools were sequenced directly to determine the actual percentage of each template (the pre-amplification in Tables S3, S4). Afterwards, we amplified the pools using the optimized system, and the percentages of each *TRBV* segment are shown as the post-amplification in Tables S3, S4. The biases representing as the differences between the post- and pre-amplification concentrations were carefully evaluated. Although the expected concentration difference between some TRBV segments was several orders of magnitude, the frequency of these TRBV segments was similar before and after amplification (Figures 2G,H), and the biases were between 0.5 and 2 (Figure S4).

### The Synthetic *TRB* Templates Can Serve as Spike-in Material to Calibrate the Residual Amplification Bias

**Figure 3:**
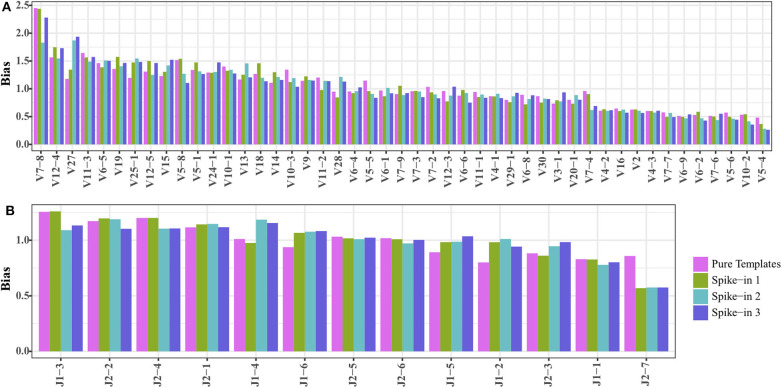
Comparing the **(A)** *TRBV* and **(B)** *TRBJ* amplification bias of *TRB* templates as spike-in and pure templates. The spike-ins 1–3 indicated that 20,000 *TRB* templates were mixed with three natural DNA samples.

We mixed 20,000 copies of *TRB* templates (pool 1) into the natural DNA samples to test if the *TRB* repertoire of natural samples would affect the amplification biases of the synthetic templates. The reads mapped to the synthetic *TRB* templates were split out after analysis using *IMonitor*, and the result showed that the amplification bias of the spike-in *TRB* templates was almost the same as that of the pure templates (Figures 3A,B). Therefore, the synthetic templates mixture can serve as a reference control to be spiked into natural samples, to evaluate or even calibrate the residual biases generated during the amplification.

### MRD Detection in T-ALL

For two T-ALL patients, we detected MRD based on the *TRB* repertoire data and then compared it with the result detected by flow cytometry (FC). We calculated the percentage of T cells in total nucleated cells using the *TRB* repertoire data, and the result was similar to that detected by FC (Table 1). For MRD detection, when the MRD level is above 1%, the detection result of TCR-seq is comparable with that of FC; when MRD level is <0.5%, the FC gives a negative result. However, TCR-seq could detect the MRD with a higher sensitivity.

**Table 1:** Comparing MRD detection results by TCR-seq and FC of T-ALL patients.

**Patients**	**DAC**	**Cancer clonotype in TCR-seq data (%)**	**T cell percentage by FC (%)**	**T cell percentage by TCR-seq (%)**	**MRD by FC (%)**	**MRD by TCR-seq (%)**
CYY	0	85.4	69	27.83	NA	NA
	33	24	12.18	14.36	3.8	3.45
	96	0.45	15.41	13.02	0	0.06
TDJ	8	63.96	ND	29.96	ND	19.16
	15	49.36	32.26	31.74	18.5	15.67
	33	2	15.16	15.30	0	0.31

The TRB CDR3 of cancer clonotype for patients CYY:

*TGCAGTGCCTCGCCTCCTCCTAGCGGGAGGGGGAATGAGCAGTTCTTC*.

The TRB CDR3 of cancer clonotype for patients TDJ:

*TGCAGTGCTAGAGACCTGGGACAGGGGAGCAGGATTAGCTCCTACGAGCAGTACTTC*.

*DAC, day after beginning of chemotherapy; NA, not applicable; ND, not detection*.

### Dynamics of *TRB* Repertoire in Patients With B-ALL During Chemotherapy

**Figure 4:**
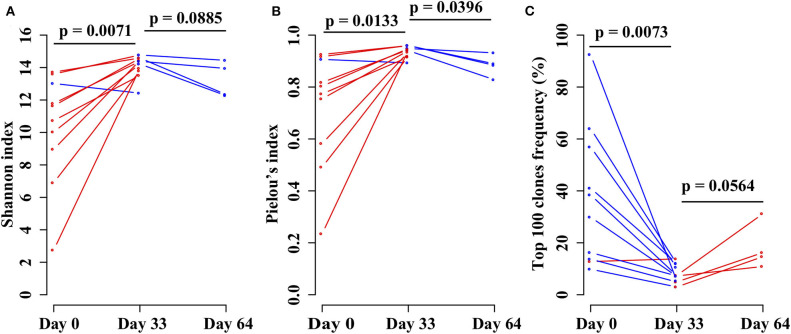
Comparing the **(A)** Shannon index, **(B)** Pielou's index, and **(C)** percentage of top 100 maximal frequency clonotypes among three different time points of the same B-ALL patients. The three time points were before chemotherapy and 33 and 64 days after chemotherapy. *P*-value was calculated by two-sided paired *t*-test.

In order to investigate the changes in the *TRB* repertoire in B-ALL patients during chemotherapy, the diversity, evenness, and clonality of the repertoire at different time points were compared. The diversity is represented by Shannon index, which reflects the richness of the repertoire and is a popular index used to measure the diversity of immune repertoire. The Shannon index increases from day 0 to 33 and then decreases from day 33 to 64 (Figure 4A). The evenness is assessed using the Pielou's index, and the same trend is identified as Shannon index (Figure 4B). The clonality is evaluated using the total frequency of top 100 *TRB* clonotypes, which can reflect the expansion of the clonotypes. In contrast to the diversity, the clonality decreases from day 0 to 33 and increases from day 33 to 64 (Figure 4C).

**Figure 5:**
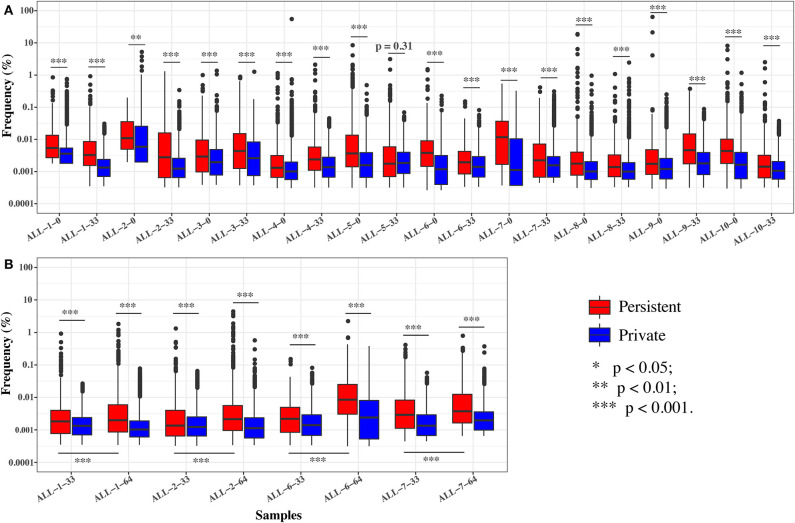
The frequencies of the persistent and private clonotypes on two time points. **(A)** On days 0 and 33. **(B)** On days 33 and 64. ALL-1-0, ALL-1-33, and ALL-1-64 refer to frequencies on days 0, 33, and 64, respectively. Paired tests were performed on the frequencies of persistent clonotypes between day 33 and 64.

**Figure 6:**
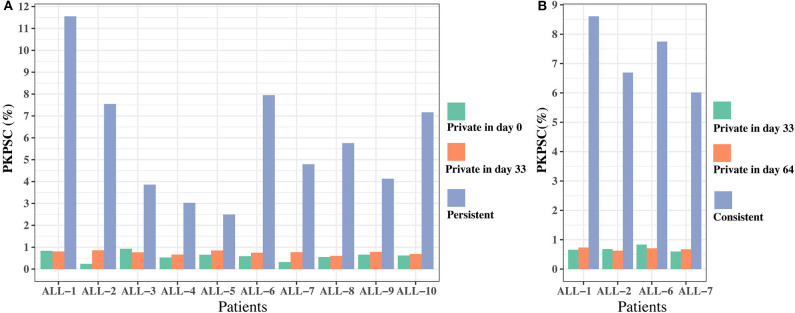
The proportion of known pathogens specific *TRB* clonotypes (PKPSC) in persistent and private clonotypes on two time points. **(A)** On days 0 and 33. **(B)** On days 33 and 64.

To further investigate the effect of chemotherapy on the dynamics of TCR clonotypes, we evaluated the frequencies and characteristics of the persistent clonotypes (overlapped clonotype between time points) from day 0 to 33 and from day 33 to 64 in the same patient. During induction treatment (days 0–33), most (95.94% on average) of the *TRB* clonotypes present on day 0 vanished on day 33, whereas a greater number (98.16% on average) of *TRB* clonotypes on day 33 were newly emerged (Table S5). For the persistent clonotypes from day 0 to 33, their frequencies in the repertoire were significantly higher than the private clonotypes (existing in only one time point) in all samples (Figure 5A). During consolidation chemotherapy treatment (days 33–64), the frequencies of the persistent clonotypes were also higher than the private ones in the repertoire and, to be noted, were significantly increased on day 64 compared to day 33 in all four patients (Figure 5B). To further explore the characteristics of the persistent clonotypes, we investigated the differences in PKPSC between the persistent and the private clonotypes. Notably, the PKPSC in persistent clonotypes was substantially higher than that in private clonotypes, indicating the enrichment of high-avidity T cells targeting known pathogens in the persistent clonotypes (Figure 6).

## Discussion

The immune system plays critical roles in human health, and a diverse TCR repertoire is necessary to warrant the potential of antigen recognition. In the future, if the antigen specificity of all the TCR CDR3s can be decoded, the T cell repertoire data will be of great help in disease diagnosis, treatment management, prognosis monitoring, and even risk evaluation. For future clinical applications, the most important thing is that we can accurately quantify the TCR repertoire. In order to evaluate the accuracy of the TCR-seq method, a TCR repertoire standard or reference with known compositions is particularly important (42); however, there are still no certified reference materials in this field. In this paper, we designed and synthesized 611 *TRB* templates, all of which have CDR3 that are out of frame to ensure that they do not exist in actual sample. We checked the *TRB* repertoire data of ~6,000 human peripheral blood samples and did not find these templates appearing in any sample. Using these templates as reference materials, we optimized our *TRB* repertoire sequencing method and minimized the bias of multiplex PCR system.

The feasibility of immune repertoire sequencing technologies in MRD detection for blood cancer has been demonstrated in many studies (17, 40, 43, 44). Wood et al. (44) also reported that the patients with an MRD level of 0.01% by clonoSEQ, but negative by FC, had worse EFS compared with the patients who had a negative MRD by clonoSEQ (44). In fact, the clonoSEQ provided by Adaptive Biotechnologies has been cleared by the Food and Drug Administration (FDA) to detect the MRD for B-ALL and multiple myeloma. In this paper, we verified that TCR-seq MRD detection is more sensitive than FC for T-ALL patients.

Previous studies have demonstrated the immune reconstitution in leukemia patients after chemotherapy (45) and during chemotherapy (46) at the cellular subpopulation level. However, it is still unknown how chemotherapy affects the TCR repertoire of B-ALL patients. By analyzing the changes in *TRB* repertoire during chemotherapy, we showed that the diversity of *TRB* repertoire increases from day 0 to 33, which may relate with the recovery of the T cell number on day 33 at the end of the induction therapy (46). Consistently, the number of clonotypes on day 33 is 2–10 times that on day 0 from our data (Table S5). These may be rare preexisting naive T cells in the periphery or newly generated and recruited T cells, which is supported by T cell receptor excision circle (TREC) assay (46). We speculate that induction therapy could promote the recruitment and stimulation of a large number of pathogen-reactive naive T cells and diversify the repertoire. During the consolidation stage from day 33 to 64, these clonotypes experience further expansion, possibly driven by infection or external environment, which lower the repertoire diversity. Our data support that some relatively abundant TCR clonotypes, which are usually antigen experienced and target pathogens, are more likely to be selected under infection or other external environmental pressures and to be stably maintained during chemotherapy. In fact, a previous study showed that although chemotherapy reduced the naive T cell number, the memory T cells is least affected and preserved (47). However, it should be noted that it is difficult to analyze how much the repertoire is changed by infection, disease regression, or other prognosis factors, especially that we do not have these clinical data.

In summary, we have designed and synthesized some *TRB* reference templates to optimize the *TRB* multiplex reaction system. We then used this experimental system to detect the MRD in T-ALL patients. We also used it to investigate the dynamic change of TRB repertoire during the chemotherapy of B-ALL and found that the pathogen-specific memory T cells are selected and expanded during chemotherapy. Chemotherapy could suppress the immune system of cancer patients and put them at risk of infection; therefore, the recovery of the immune system during and after chemotherapy is very critical for cancer patients to resist infection and other immune related disease. Considering the importance of antigen recognition potential of immune system, in the future, combined with other omics technologies, it is possible that precise immune repertoire data could provide great help in evaluating an individual's immunity.

## Data Availability Statement

The data reported in this study are available in the CNGB Nucleotide Sequence Archive (CNSA: https://db.cngb.org/cnsa; accession number CNP0000882).

## Ethics Statement

The studies involving human participants were reviewed and approved by BGI-IRB. Written informed consent to participate in this study was provided by the participants' legal guardian/next of kin.

## Author Contributions

SL, XiaW, HM, XY, and FW collected the samples and clinical information. JW, LLi, XieW, and MF performed the experiments. XuL, WZ, LLu, YZ, and ZW performed the data analysis and visualization. JW, CL, and XiL analyzed and interpreted the data. JW wrote the manuscript. CL and XiL supervised the study. All authors contributed to the article and approved the submitted version.

## Conflict of Interest

JW, XieW, LLi, XuL, WZ, LLu, ZW, MF, YZ, and XiL were employed by BGI-Shenzhen. The remaining authors declare that the research was conducted in the absence of any commercial or financial relationships that could be construed as a potential conflict of interest.

## Abbreviations

TCR: T cell receptor
TCR-seq: TCR repertoire sequencing
PCR: polymerase chain reaction
TRB: TCR beta chain
ALL: acute lymphoblastic leukemia
B-ALL: B-cell acute lymphoblastic leukemia
T-ALL: T-cell acute lymphoblastic leukemia
MRD: minimal residual disease
CDR3: complementarity determining region 3
PKPSC: proportion of known pathogen-specific *TRB* clonotypes
FC: flow cytometry.
