# Supplementary material for: Developing an Unbiased Multiplex PCR System to Enrich the TRB Repertoire Toward Accurate Detection in Leukemia
Source: Front Immunol. 2020 Aug 6;11:1631. doi: 10.3389/fimmu.2020.01631 (PMC7423970; doi:10.3389/fimmu.2020.01631)
Supplement: Supplementary file 1 [file Data_Sheet_1.docx]

Supplementary Material

1. **Supplementary Figures and Tables**

## Supplementary Figures

**Figure S1.** The relative percentages of each of the 611 *TRB* templates before amplification in pool 1 (A) and pool 2 (B).

**Figure S2.** The *TRBV* (A, C) and *TRBJ* segments (B, D) usage frequency before amplification of pool 1 (A, B) and pool 2 (C, D).

**Figure S3.** Amplification bias of the multiplex PCR reaction system during primer mix optimization for pool 2. (A) The *TRBV* amplification bias before optimization. (B, C) The *TRBV* amplification bias during optimization. (D) The *TRBV* amplification bias after optimization. (E) The *TRBJ* amplification bias before optimization. (F, G) The *TRBJ* amplification bias during optimization. (H) The *TRBJ* amplification bias after optimization.

**Figure S4.** Amplification biases for the first (A) and second (B) gradient mixture of the TRB templates

## Supplementary tables

**Table S1.** Primers used in multiplex PCR for amplifying the CDR3 of rearranged *TRB* gene.

| TRBV Primers | Primer Sequence |
| --- | --- |
| zeTRBV1 | GACCGCTTGGCCTCCGACTTATTTCACTCTGAAGATCCGGTCCAC |
| zeTRBV2-new | GACCGCTTGGCCTCCGACTTGCTTGGTGACTCTGCTGTGTATTTC |
| zeTRBV3 | GACCGCTTGGCCTCCGACTTCAAGTCGCTTCTCACCTGAATG |
| zeTRBV4 | GACCGCTTGGCCTCCGACTTGCCAGTTCTCTAACTCTCGCTCT |
| zeTRBV5 | GACCGCTTGGCCTCCGACTTTCAGGTCGCCAGTTCCCTAAYTAT |
| zeTRBV6 | GACCGCTTGGCCTCCGACTTCACGTTGGCGTCTGCTGTACCCT |
| zeTRBV7 | GACCGCTTGGCCTCCGACTTCAGGCTGGTGTCGGCTGCTCCCT |
| zeTRBV10 | GACCGCTTGGCCTCCGACTTGGGATCCGTCTCCACTCTGAMGAT |
| zeTRBV11 | GACCGCTTGGCCTCCGACTTGGGATCCGTCTCTACTCTGAAGAT |
| zeTRBV12 | GACCGCTTGGCCTCCGACTTGGGATCTTTCTCCACCTTGGAGAT |
| zeTRBV13 | GACCGCTTGGCCTCCGACTTCCTGACTTGCACTCTGAACTAAACCT |
| zeTRBV14 | GACCGCTTGGCCTCCGACTTCCTCACTCTGGAGTCTGCTGCC |
| zeTRBV15 | GACCGCTTGGCCTCCGACTTCCTCACTCTGGAGTCMGCTACC |
| zeTRBV16 | GACCGCTTGGCCTCCGACTTGCAGAGAGGCTCAAAGGAGTAGACT |
| zeTRBV17 | GACCGCTTGGCCTCCGACTTGAAGATCCAGCCCTCAGAACCCAG |
| zeTRBV18 | GACCGCTTGGCCTCCGACTTTCGATTCTCAGCTCAACAGTTC |
| zeTRBV19 | GACCGCTTGGCCTCCGACTTGGAGGGACGTATTCTACTCTGAAGG |
| zeTRBV20 | GACCGCTTGGCCTCCGACTTTTCTTGACATCCGCTCACCAGG |
| zeTRBV21 | GACCGCTTGGCCTCCGACTTCTGTAGCCTTGAGATCCAGGCTACGA |
| zeTRBV22 | GACCGCTTGGCCTCCGACTTTAGATGAGTCAGGAATGCCAAAG |
| zeTRBV23 | GACCGCTTGGCCTCCGACTTCTGTGACATCGGCCCAAAAGAAC |
| zeTRBV24 | GACCGCTTGGCCTCCGACTTAACCATGCAAGCCTGACCTT |
| zeTRBV25 | GACCGCTTGGCCTCCGACTTCTCCCTGTCCCTAGAGTCTGCCAT |
| zeTRBV26 | GACCGCTTGGCCTCCGACTTGCCCTCACATACCTCTCAGTACCTC |
| zeTRBV27 | GACCGCTTGGCCTCCGACTTGATCCTGGAGTCGCCCAGC |
| zeTRBV28 | GACCGCTTGGCCTCCGACTTATTCTGGAGTCCGCCAGC |
| zeTRBV29 | GACCGCTTGGCCTCCGACTTAACTCTGACTGTGAGCAACATGAG |
| zeTRBV30-new | GACCGCTTGGCCTCCGACTTTCCTTCTCAGTGACTCTGGCTTCTATC |
| TRBJ primers | Primer Sequence |
| zeTRBJ1 | ACATGGCTACGATCCGACTTCTTACCTACAACTGTGAGTCTGGTG |
| zeTRBJ2 | ACATGGCTACGATCCGACTTACCCCCAGCCTTACCTACA |
| zeTRBJ3 | ACATGGCTACGATCCGACTTCTTACCTACAACAGTGAGCCAACTT |
| zeTRBJ4 | ACATGGCTACGATCCGACTTAAGACAGAGAGCTGGGTTCCACT |
| zeTRBJ5 | ACATGGCTACGATCCGACTTCTTACCTAGGATGGAGAGTCGAGTC |
| zeTRBJ6 | ACATGGCTACGATCCGACTTCGAGTCAAGAGTGGAGCCC |
| zeTRBJ7 | ACATGGCTACGATCCGACTTCCTTCTTACCTAGCACGGTGA |
| zeTRBJ8 | ACATGGCTACGATCCGACTTCTTACCCAGTACGGTCAGCCT |
| zeTRBJ9 | ACATGGCTACGATCCGACTTCCGCTTACCGAGCACTGTCAG |
| zeTRBJ10 | ACATGGCTACGATCCGACTTAGCACTGAGAGCCGGGTCC |
| zeTRBJ11 | ACATGGCTACGATCCGACTTCGAGCACCAGGAGCCGCGT |
| zeTRBJ12 | ACATGGCTACGATCCGACTTCTCGCCCAGCACGGTCAGCCT |
| zeTRBJ13 | ACATGGCTACGATCCGACTTCTTACCTGTGACCGTGAGCCTG |

**Table S2. The clinical information of the leukemia patients**

| Patients ID | Disease | Age | Gender | Risk group | Fusion gene | MRD on day 33 | MRD on day 64 | Clinical outcome |
| --- | --- | --- | --- | --- | --- | --- | --- | --- |
| ALL-1 | B-ALL | 2 | M | SR | Normal | 0 | 0 | CR |
| ALL-2 | B-ALL | 5 | F | SR | TEL-AML1 (+) | 0 | 0 | CR |
| ALL-3 | B-ALL | 8 | M | IR | Normal | 0 |  | CR |
| ALL-4 | B-ALL | 10 | F | IR | TEL-AML1 (+) | 0 |  | CR |
| ALL-5 | B-ALL | 1 | M | HR | MLL (+) | 0 |  | CR |
| ALL-6 | B-ALL | 2 | F | SR | Normal | 0 | 0 | CR |
| ALL-7 | B-ALL | 4 | M | SR | TEL-AML1 (+) | 0 | 0 | CR |
| ALL-8 | B-ALL | 2 | F | SR | TEL-AML1 (+) | 0 |  | CR |
| ALL-9 | B-ALL | 10 | M | HR | TEL-AML1 (+) | 0.6% |  | Unknown |
| ALL-10 | B-ALL | 3 | M | IR | TEL-AML1 (+) | 0.3% |  | CR |
| CYY | T-ALL | 11 | M | IR | Normal | 3.8% |  | Death  after bone marrow transplantation |
| TDJ | T-ALL | 4 | M | IR | Not done | 0 |  | CR after bone marrow transplantation |

R = Standard Risk; IR = Intermediate Risk; HR = high risk; CR = complete remission

**Table S3.** The expected, pre-amplification and post-amplification values for each TRBV segment of the first pool of 47 TRBV segments with different concentrations. The red color indicated the TRBV segments with gradient pooling.

| TRBV segments | expected | pre-amplification | post-amplification | bias |
| --- | --- | --- | --- | --- |
| V11-2 | 20 | 19.4754 | 16.7904 | 0.862 |
| V11-3 | 0.1 | 0.0856 | 0.0906 | 1.059 |
| V12-4 | 10 | 8.8974 | 11.8737 | 1.335 |
| V18 | 5 | 3.2469 | 7.0525 | 2.172 |
| V19 | 0.01 | 0.0089 | 0.0077 | 0.866 |
| V20-1 | 0.1 | 0.0957 | 0.0922 | 0.963 |
| V25-1 | 0.01 | 0.0088 | 0.0044 | 0.503 |
| V29-1 | 1 | 1.1062 | 1.1572 | 1.046 |
| V30 | 1 | 1.2363 | 0.6039 | 0.488 |
| V9 | 0.05 | 0.0449 | 0.0277 | 0.616 |
| V10-1 | 1.695 | 2.0043 | 2.9619 | 1.478 |
| V10-2 | 1.695 | 2.0942 | 1.0390 | 0.496 |
| V10-3 | 1.695 | 1.5912 | 1.4641 | 0.920 |
| V11-1 | 1.695 | 1.6642 | 2.0053 | 1.205 |
| V12-3 | 1.695 | 1.6602 | 1.8728 | 1.128 |
| V12-5 | 1.695 | 1.3689 | 1.9119 | 1.397 |
| V13 | 1.695 | 2.3374 | 2.5496 | 1.091 |
| V14 | 1.695 | 2.1300 | 2.8583 | 1.342 |
| V15 | 1.695 | 1.6489 | 2.8491 | 1.728 |
| V16 | 1.695 | 1.9802 | 0.9207 | 0.465 |
| V2 | 1.695 | 2.1288 | 1.1575 | 0.544 |
| V24-1 | 1.695 | 1.4965 | 1.1986 | 0.801 |
| V27 | 1.695 | 2.4446 | 2.5119 | 1.028 |
| V28 | 1.695 | 1.8385 | 1.3498 | 0.734 |
| V3-1 | 1.695 | 1.8930 | 0.6653 | 0.351 |
| V4-1 | 1.695 | 2.4844 | 1.0772 | 0.434 |
| V4-2 | 1.695 | 1.6215 | 0.8815 | 0.544 |
| V4-3 | 1.695 | 1.4234 | 0.8602 | 0.604 |
| V5-1 | 1.695 | 2.1375 | 2.9725 | 1.391 |
| V5-4 | 1.695 | 1.4855 | 0.6134 | 0.413 |
| V5-5 | 1.695 | 1.1276 | 1.6230 | 1.439 |
| V5-6 | 1.695 | 1.4410 | 0.9922 | 0.689 |
| V5-8 | 1.695 | 1.6262 | 3.1391 | 1.930 |
| V6-1 | 1.695 | 1.7295 | 1.2677 | 0.733 |
| V6-2 | 1.695 | 1.7845 | 1.0453 | 0.586 |
| V6-4 | 1.695 | 1.5745 | 1.6246 | 1.032 |
| V6-5 | 1.695 | 1.4956 | 1.6371 | 1.095 |
| V6-6 | 1.695 | 1.7689 | 1.0540 | 0.596 |
| V6-8 | 1.695 | 1.7901 | 1.8489 | 1.033 |
| V6-9 | 1.695 | 1.7762 | 1.1731 | 0.660 |
| V7-2 | 1.695 | 2.1106 | 2.4326 | 1.153 |
| V7-3 | 1.695 | 1.7253 | 2.4855 | 1.441 |
| V7-4 | 1.695 | 1.2211 | 1.1583 | 0.949 |
| V7-6 | 1.695 | 1.4123 | 0.9038 | 0.640 |
| V7-7 | 1.695 | 1.3106 | 0.8203 | 0.626 |
| V7-8 | 1.695 | 1.6617 | 5.2251 | 3.144 |
| V7-9 | 1.695 | 2.8050 | 0.1486 | 0.053 |

**Table S4.** The expected, pre-amplification and post-amplification values for each TRBV segment of the second pool of 47 TRBV segments with different concentrations. The red color indicated the TRBV segments with gradient pooling.

| TRBV segments | Expected | Pre-amplification | Post-amplification | Bias |
| --- | --- | --- | --- | --- |
| V10-3 | 0.05 | 0.0439 | 0.0365 | 0.831 |
| V11-2 | 0.1 | 0.0895 | 0.0517 | 0.578 |
| V11-3 | 1 | 0.8329 | 0.6689 | 0.803 |
| V12-3 | 10 | 8.7331 | 11.9051 | 1.363 |
| V12-5 | 0.01 | 0.0176 | 0.0108 | 0.613 |
| V18 | 0.001 | 0.0037 | 0.0033 | 0.896 |
| V20-1 | 0.1 | 0.0934 | 0.0592 | 0.634 |
| V27 | 5 | 7.1276 | 11.6379 | 1.633 |
| V28 | 0.5 | 0.6248 | 0.5704 | 0.913 |
| V29-1 | 0.5 | 0.5200 | 0.4837 | 0.930 |
| V10-1 | 2.24 | 2.5580 | 4.6907 | 1.834 |
| V10-2 | 2.24 | 2.5833 | 1.5103 | 0.585 |
| V11-1 | 2.24 | 2.0662 | 2.2348 | 1.082 |
| V12-4 | 2.24 | 2.2287 | 3.4767 | 1.560 |
| V13 | 2.24 | 3.1439 | 2.5423 | 0.809 |
| V14 | 2.24 | 2.6529 | 2.9335 | 1.106 |
| V15 | 2.24 | 2.1081 | 3.4987 | 1.660 |
| V16 | 2.24 | 2.1724 | 1.2626 | 0.581 |
| V19 | 2.24 | 1.7107 | 2.4636 | 1.440 |
| V2 | 2.24 | 2.5651 | 1.5428 | 0.601 |
| V24-1 | 2.24 | 1.8134 | 1.8990 | 1.047 |
| V25-1 | 2.24 | 2.2356 | 3.7341 | 1.670 |
| V30 | 2.24 | 2.7498 | 1.8580 | 0.676 |
| V3-1 | 2.24 | 2.4507 | 1.8248 | 0.745 |
| V4-1 | 2.24 | 2.7526 | 1.0485 | 0.381 |
| V4-2 | 2.24 | 1.9369 | 0.8907 | 0.460 |
| V4-3 | 2.24 | 1.7397 | 0.8525 | 0.490 |
| V5-1 | 2.24 | 2.7568 | 3.0594 | 1.110 |
| V5-4 | 2.24 | 1.8297 | 0.4944 | 0.270 |
| V5-5 | 2.24 | 1.3699 | 1.4156 | 1.033 |
| V5-6 | 2.24 | 1.5478 | 0.8830 | 0.570 |
| V5-8 | 2.24 | 2.0762 | 3.1049 | 1.496 |
| V6-1 | 2.24 | 2.1709 | 1.6522 | 0.761 |
| V6-2 | 2.24 | 2.2966 | 1.3727 | 0.598 |
| V6-4 | 2.24 | 1.9500 | 2.1216 | 1.088 |
| V6-5 | 2.24 | 1.8812 | 2.3763 | 1.263 |
| V6-6 | 2.24 | 2.1565 | 1.3804 | 0.640 |
| V6-8 | 2.24 | 2.2405 | 2.9296 | 1.308 |
| V6-9 | 2.24 | 2.2858 | 1.8737 | 0.820 |
| V7-2 | 2.24 | 2.7227 | 2.4297 | 0.892 |
| V7-3 | 2.24 | 2.2913 | 2.6220 | 1.144 |
| V7-4 | 2.24 | 1.5622 | 1.0400 | 0.666 |
| V7-6 | 2.24 | 1.9350 | 1.0986 | 0.568 |
| V7-7 | 2.24 | 1.7203 | 0.7716 | 0.449 |
| V7-8 | 2.24 | 2.0960 | 3.0638 | 1.462 |
| V7-9 | 2.24 | 3.5444 | 0.1704 | 0.048 |
| V9 | 2.24 | 2.0123 | 2.4489 | 1.217 |

**Table S5.** The number of CDR3 nucleotide clonotypes on day 0, day 33 and the consistent clonotypes on day 0 and 33.

| Patients | No. of clonotypes on day 0 | No. of clonotypes on day 33 | No. of shared clonotypes on day 0 and 33 | Percentage of vanished pre-existing clonotypes after treatment (%) | Percentage of emerging clonotypes on day 33 (%) |
| --- | --- | --- | --- | --- | --- |
| ALL-1 | 17334 | 49265 | 374 | 97.84 | 99.24 |
| ALL-10 | 20450 | 53152 | 602 | 97.06 | 98.87 |
| ALL-2 | 3107 | 41337 | 53 | 98.29 | 99.87 |
| ALL-3 | 20635 | 12777 | 572 | 97.23 | 95.52 |
| ALL-4 | 19760 | 35898 | 1240 | 93.72 | 96.55 |
| ALL-5 | 14392 | 26429 | 1136 | 92.11 | 95.70 |
| ALL-6 | 33870 | 41303 | 466 | 98.62 | 98.87 |
| ALL-7 | 9425 | 39394 | 381 | 95.96 | 99.03 |
| ALL-8 | 23102 | 54891 | 622 | 97.31 | 98.87 |
| ALL-9 | 3025 | 28154 | 263 | 91.31 | 99.07 |
| **Average** |  |  |  | **95.94** | **98.16** |
